# Mosquito survival from mark–recapture studies releasing at known age

**DOI:** 10.1186/s13071-025-07024-2

**Published:** 2025-11-10

**Authors:** Justin Matthews

**Affiliations:** University of Exeter Medical School, St Luke’s Campus, Heavitree Road, Exeter, EX1 2LU UK

**Keywords:** Longevity, Mark–recapture, Mosquito, Senescence, Survival

## Abstract

**Background:**

The expected lifetime (EL) of mosquitoes has been the subject of a large body of historical research, including by mark–recapture (MR) methods. Previous researchers have presented collections of information informally, and the results of a large systematic search are available; however, a formal synthesis of information has not been carried out.

**Methods:**

Mosquito studies were systematically selected and re-analysed, and the information pooled to characterise an age-dependent survival model. MR data were obtained from the subset of historical studies in which mosquitoes were released at known ages after emergence. Data were excluded if experiments held characteristics, established by simulation, in which age-dependent estimation would perform poorly. Analysis used the Cormack–Jolly–Seber (CJS) model with age-dependent (Weibull) survival and time-independent capture probabilities.

**Results:**

Shape parameter estimates in many studies showed evidence of increasing mortality with age (senescence, $\alpha >1$) but also decreasing mortality ($\alpha <1$) in some, perhaps owing to high mortality early on. The expected lifetime (EL) of females was meta-analysed by the inverse-variance method taking into account parameter uncertainty, giving an overall estimate of 6.68 (95% confidence level [CL] 5.71–7.81) days and genus-specific estimates for *Aedes* (7.92, 95% CL 5.57–11.2; *n* = 5), *Anopheles* (3.61, 95% CL 2.51–5.18; *n* = 4) and *Culex* (7.22, 95% CL 6.26–8.33; *n* = 18).

**Conclusions:**

The study synthesised EL for females overall and for three important mosquito genera, taking account of mosquito age, and systematically restricting to studies with the most acceptable characteristics. The estimated quantities for *Aedes* and *Anopheles* require further caution because the number of datasets, after exclusions, is small. A number of studies were excluded on the grounds of uncertain reliability when analysed independently, and it is suggested that a future integrated (Bayesian) analysis could offer an important advantage by utilising such studies, which include older release cohorts. Increased study availability could allow further adjustment for study characteristics, notably spatial configuration.

**Graphical Abstract:**

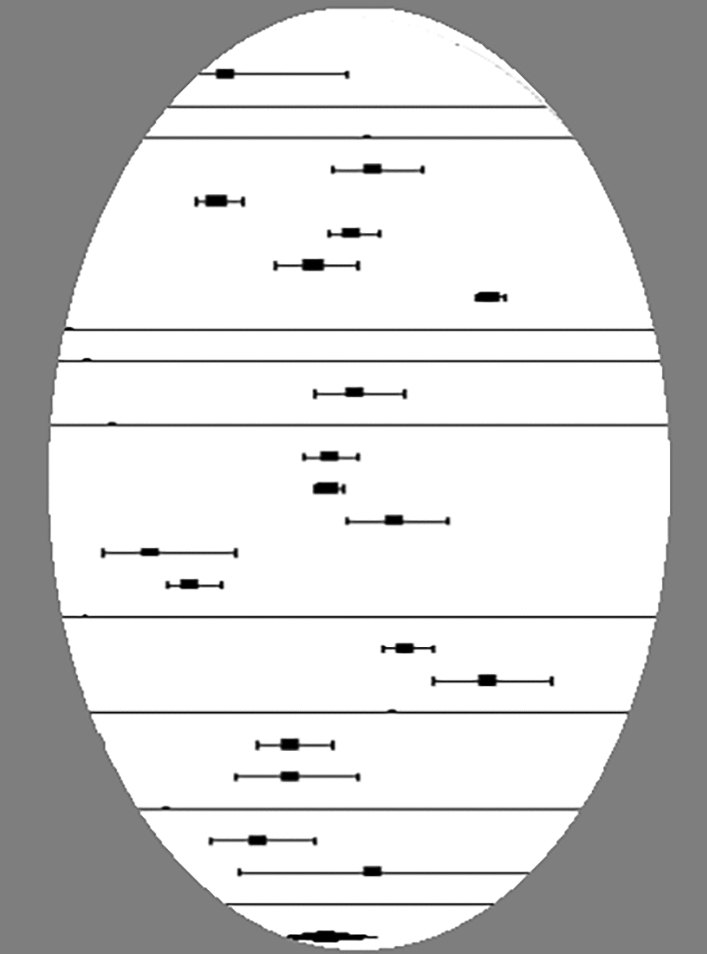

**Supplementary Information:**

The online version contains supplementary material available at 10.1186/s13071-025-07024-2.

## Background

The vector role of the mosquito in a number of infectious diseases (e.g. filariasis, malaria and yellow fever), and within this the perceived importance of understanding and controlling mosquito longevity, has led to an extensive research effort on this topic, notably in the second half of the twentieth century. The establishment of epidemiological theory (the Ross–Macdonald model and its variants) that showed the sensitivity of disease transmission to mosquito longevity has been documented by Smith et al. [1].

There was a long-standing assumption that mortality is age-independent. The underlying reasoning [2] was that death usually occurs accidentally over mosquito lifetime and is likely to occur earlier than death by senescence (deterioration with age), if there is senescence at all. It also makes for mathematical convenience, because lifetime is modelled by the exponential distribution. Then the hazard rate is constant, and mortality or survival may be modelled with a single parameter. Furthermore, the mortality of a mosquito at any time or age is independent of the past, so the future expected lifetime (EL) of the mosquito vector is constant for a mosquito, regardless of age.

Evidence has accumulated in more recent times that this age-independent assumption may be too reductive. In the laboratory, external/wild sources of mortality are excluded, and Styer et al. [3] used large-scale laboratory information (> 100,000 mosquitoes) finding evidence of senescence, in which models with accelerating mortality were a superior fit to the exponential. For *Anopheles* mosquitoes, the authors of Dawes et al. [4] also presented results showing that life expectancy was dependent on age (and also on *Plasmodium* infection density). For laboratory data, the authors of Brady et al. [5] selected generalised additive models on the basis of ‘more accurate quantification of mortality rate and its improved fit across a range of temperatures’ and concluded that fitting indicated the presence of age-dependent survival. In the wild, Clements and Paterson [6] re-examined several past studies with regard to their age-structure and concluded that in some cases the evidence favoured an age-dependent (Gompertz) model. A logistic regression for recapture by Harrington et al. [7] reported a significant decline in recapture with age.

A small number of reviews or syntheses of this body of information on mosquito survival have been carried out including those just mentioned, along with other informal presentations (e.g. [8], Supplementary Table S3). Many of these were carried out before the advent of methodology that advances and applies a systematic approach to collation of information, so the search strategies, exclusion criteria and so on were not recorded. A systematic search was carried out by Guerra et al. [9], which collated information and provided a database containing study characteristics; however, that work stopped short of a synthesis. More recently, the authors of Matthews et al. [10] carried out a synthesis centred on the broad methodology (whether using a mark–recapture, age-structure or parasitological approach) but working with crude summary data from the original publications (in particular, probability of daily survival). It appears there has been no study to date that marries systematic and reasonably comprehensive searching with an up-to-date statistical analysis method.

Of the suite of methods used for estimating mosquito survival, Matthews et al. [10] argue that mark–recapture is the most empirically grounded. No assumptions of stable age structure or assumed (or estimated) population growth rate need be made, and more systematic and extensive data are often recorded. The approach has been used extensively with mosquitoes: the authors of Guerra et al. [9] report 161 source studies using mark–recapture (MR). Mosquitoes are marked, most commonly by paint (but also more novel means such as radioactive material or bacterial infection), they are released and over the subsequent period some are recaptured. A readable explanation of what was and remained the conventional form of analysis is given by Milby and Reisen [11], using a regression of transformed counts of recaptures, with an assumption of age-independent survival (see Ref. [12] for improvements).

In this paper, we extend the evidence base by collecting and analysing a wider body of MR studies than has previously been examined. The database from Guerra et al. [9] strongly informed our study, providing an invaluable body of references and information on study characteristics, notably recording ages of released mosquitoes when known. Our study has utilised the original MR data rather than summary information. The source studies were largely not conceived or analysed with age-dependent survival at the time but nonetheless can provide data towards it. We have collated MR information from historical studies and reanalysed them with a Weibull survival model that does not make the conventional assumption of age-independence (though it includes it as a special case).

The particular MR studies used follow cohort(s) of mosquitoes of known age at release (marked for example with paint or dust) and examine the pattern of recaptures to assess mortality. The age at release is usually controlled by collecting larvae either in the wild or from mosquitoes raised in insectaries and tracking their age from emergence until marked release. This is in contrast to another historically common study type in which adult mosquitoes are collected and marked before re-release; the age of these individuals is likely unknown.

Our methodological approach is briefly as follows: We fitted survival parameters to data from historical mark–recapture studies which released mosquitoes at known age or where age at release could be surmised. We fitted Cormack–Jolly–Seber (CJS) models applying the likelihood equation supplied by McRea and Morgan [13], allowing for age-dependence in survival, with time-independent capture probability. The survival rates were determined by a Weibull survival model with two parameters which describe shape and scale. The parameters are estimated by study and presented along with corollary information on EL, and the average expected lifetime of mosquitoes overall and in the genera *Aedes*, *Anopheles* and *Culex* is estimated by meta-analysis.

## Methods

### Scoping

A scoping exercise was carried out to assess the feasibility of a synthesis of MR studies with respect to survival. A query of the Guerra et al. [9] database indicated the number of mark–recapture studies with or without known-age releases, which is presented in Table 1. Though known-age studies are a minority, 51 were available with potential information. The set of ages-at-release in any study was usually small: single-age release, more occasionally two or three. A prime requirement was that MR data were available that could be put into the form of a capture history matrix or m-array (these data structures are explained further in the section hereafter). Scoping found that publications never reported the capture history matrix and rarely the full m-array but often reported m-array information directly in a tabular form or indirectly in graphical form. Single-release experiments were much more common and would usually yield information for a single-row m-array. Recapture was usually carried out with similar apparatus and effort from occasion to occasion. Mosquitoes were usually killed on recapture; experiments with re-release of recaptured mosquitoes were very unusual. The exercise concluded that there were no strong obstacles to a pooled analysis of studies making use of an age-dependent CJS model, and the assumption of time-independent capture probability was defensible.

**Table 1:** Counts of MR studies with or without known-age release from Ref. [9]

Age	*Aedes*	*Anopheles*	*Culex*
Known	24	11	16
Unknown	30	70	28

### Data

The ‘capture history’ of the cohort(s) can be put in matrix form. This mark–recapture matrix forms a summary of the set of individual capture histories in the experiment, of which there are ${2}^{T}-1$ possibilities where *T* is the number of recapture occasions. Under the assumptions of mark–recapture, an even more concise summary is provided by the ‘reduced m-array’ (henceforth ‘m-array’), which counts the numbers of mosquitoes released at *i* and next caught at *j*, without regard to the capture history prior to *i* or subsequent to *j*. The m-array is the usual form of reporting recapture data in publications.

When only a single release is carried out, the data reported can be structured as a single-row m-array, and this is the most common format. In some experiments, the release and/or recapture occasions are irregularly spaced.

An example m-array is shown here from Takagi et al. [14], in which three cohorts of 4-day old marked mosquitoes were released on three successive days (days 22, 23 and 24):

**Table:** 

23	24	25	26	27	28	29	30	31	32	33	34	35	36	37	Uncaptured
1	4	14	2	1	12	5	6	2		1	3		0	4	241
	1	2	0	0	6	3	3	1		0	0		1	0	144
		7	0	1	3	2	5	1		1	0		0	0	229

The first to penultimate columns show a set of counts ${m}_{ij}$ of animals released at occasion *i* and subsequently caught at occasion *j*. The final column shows the numbers released but not re-caught again over the course of the experiment. In this example, the final column was calculated from the supplied numbers released and numbers re-caught. No recaptures were attempted on days 32 and 35, so the columns are empty.

A much fuller expression of the study information contains m-arrays by age (the ‘full’ or ‘generalised’ m-array, McRea and Morgan [13]). This structure is a set of counts with ${m}_{ij}\left(a\right)$ denoting the number of individuals which, when released on day *i* at age *a* days, are next captured at *j*. Accompanying it is an array ${R}_{i}\left(a\right)$, which is the number of individuals released on day *i* of age *a* days. The generalised m-array is almost never reported directly but could in principle be surmised from the study report. A reduced m-array for an experiment with cohorts of known age can be expressed as a generalised m-array.

In the example above, the cohorts released were all of the same age on each occasion (multiple release, single age). An alternative is to release multiple ages simultaneously (single release, multiple ages), for which a full m-array data structure is required. Harrington et al. [15] simultaneously released ‘young’ and ‘old’ cohorts (3 and 13 days old, respectively) in Puerto Rico. The data can be formed to give the following R-array.

**Table:** 

Age	1
3	142
13	122

and generalised m-array:

**Table:** 

Age	2	3	4	5	6	7	8	9	10	11	Uncaptured
3	20	15	9	0	3	0	1	1	0	1	92
13	13	3	1	1	0	0	0	0	1	0	103

Finally, different ages may be released at each occasion (single or multiple release and multiple age). For example, Eldridge and Reeves [16] released cohorts of ages 5, 1 and 2 on days 1, 4 and 7, respectively.

The MR and release data were extracted from the original studies and then entered into R as arrays.

A study may report more than one MR experiment, with releases separated in time and space. For example, Reisen et al. [17] reported separate experiments carried out in different months. When release cohorts overlap, for our study, a multiple-release m-array was formed where possible. Recaptures from separate releases were sometimes accumulated in the source publication in such a way that they could not be disaggregated and had to be treated as a single-release experiment, e.g. in Ref. [18].

Other aggregation or conditioning factors include experimental site, sex, mosquito species and age. For example, Reisen et al. [17] released and recaptured male and female cohorts of *Cu. tarsalis* and *Cu. quinquefasciatus*. However, the recapture data were only reported fully for *Cu. tarsalis* females. Table 2 presents a brief summary of study and dataset-level characteristics.

**Table 2:** Summary of included study characteristics

Study	Dataset details	Study genus	Study species	Number of ages released	Number of release occasions	Number of recapture occasions	Total released	Total recaptured
Beier 1982		*Aedes*	*triseriatus*	1	1	15	3734	254
Gillies 1961		*Anopheles*	*gambiae*	1	1	26	202,200	1021
Jensen 1991		*Aedes*	*melanimon*	1	1	15	15,487	136
Jensen [24]		*Aedes*	*vexans*	1	1	15	3231	63
Lindquist 1967		*Culex*	*pipiens*	1	1	10	140,000	83
Liu 2012	Study site II (newly emerged)	*Anopheles*	*sinensis*	1	1	9	1500	33
Midega 2007	Mtepeni village	*Anopheles*	*gambiae and funestus*	1	1	18	1246	59
Milby [11]		*Culex*	*quinquefasciatus*	1	1	13	3710	371
Nayar [28]	Experiment 2	*Culex*	*nigripalpus*	1	1	12	25,000	422
Nayar [28]	Experiment 3	*Culex*	*nigripalpus*	1	1	12	35,000	430
Nayar [28]	Experiment 4	*Culex*	*nigripalpus*	1	1	12	35,000	548
Nayar [28]	Experiment 5	*Culex*	*nigripalpus*	1	1	12	60,000	1540
Nelson [23]	August	*Culex*	*tarsalis*	1	1	10	3000	183
Nelson [23]	July	*Culex*	*tarsalis*	1	1	10	2250	93
Nelson [23]	June	*Culex*	*tarsalis*	1	1	10	1500	97
Nelson [23]	September	*Culex*	*tarsalis*	1	1	10	1710	79
Nelson 1982		*Culex*	*tarsalis*	2	1	11	7428	393
Nelson 1982		*Culex*	*tarsalis*	2	1	11	7185	541
Quiroga 2006		*Culex*	*quinquefasciatus*	1	2	12	2352	110
Qurashi 1966		*Anopheles*	*stephensi*	1	1	9	58,500	162
Reisen 1979b		*Anopheles*	*stephensi*	1	3	9	10,119	379
Reisen [18]		*Anopheles*	*culicifacies*	1	1	13	1908	153
Reisen [17]	August	*Culex*	*tarsalis*	1	1	12	12,631	321
Reisen [17]	July	*Culex*	*tarsalis*	1	1	14	17,948	292
Reisen [17]	June	*Culex*	*tarsalis*	1	1	11	8505	64
Reisen [17]	May	*Culex*	*tarsalis*	1	1	10	14,050	82
Reisen [17]	September	*Culex*	*tarsalis*	1	1	9	2539	44
Trpis 1995		*Aedes*	*aegypti*	1	1	9	2000	338
Watson 2000		*Aedes*	*notoscriptus*	1	2	8	1242	88
Yamar 2005	Neonates	*Aedes*	*vexans*	1	1	12	13,600	34

### Searching

The references identified by Guerra et al. [9] were supplemented by a much smaller set of references collected ad hoc by the author (*n* = 26), and combined with a Web of Science search from 2014 to date [title terms: mosquito AND (surviv* OR longevity OR mortality)] to create the complete reference set, *n* = 188.

A flow diagram of the search and selection process is provided in Additional File 1: Supplementary Fig. S1 (Appendix S2). Studies in which a survival-related experimental negative intervention (e.g. genetic modification) was apparent from the title were excluded. Studies in which age of release was unknown were excluded. For example, Takken et al. [19] captured mosquitoes from houses with aspirators prior to marking, so the ages of these adults at release were not known. Studies without useable MR information were excluded. For example, the authors of Marini et al. [20] reported recapture numbers in aggregated form, e.g. in the first experiment as 2–5, 6–9 and 10–21 day totals; these data could not be put into the usual m-array form by recapture day, and the study was therefore excluded.

None of the selected datasets were exclusively concerned with males, and the majority contained female-specific MR information, so the analysis followed Ref. [9] in confining results to females. Similarly, the vast majority of datasets were in the three genera *Anopheles*, *Aedes* and *Culex*; other genera were excluded.

Studies were then filtered according to conditions established by simulation (details below). For example, Takagi et al. [14] released three laboratory-reared cohorts 4–5 days after emergence. Criteria established on the basis of simulation results excluded this single-age study because of the older age of the mosquito cohorts and the inadequate size (< 500) of the release cohorts (296, 161 and 249).

After exclusions, there were 73 MR datasets with ages known at release and, from these, 30 datasets of female mosquitoes with suitable MR information and experimental characteristics. The references supplying the final datasets are listed in Additional File 2.

### Analysis

After the selection of studies described above, analysis is carried out in two stages. In the first stage, the parameters of a mark recapture model are estimated for each selected study, which include survival and capture parameters. The capture parameters have a modelling function, but the survival parameters are of primary interest. Study-specific capture probabilities are ascribed to each study, allowing study characteristics (experimental design, local conditions, etc.) to influence the data. For example, a study in which recapture uses baited recapture is allowed a different (probably higher) recapture probability to one that does not. In this way, important heterogeneity is modelled. In the second stage, the EL and its variance are estimated from the survival parameters, and this outcome is analysed by conventional meta-analysis.

In the first stage, each study is analysed using the CJS model. In our analysis, the Weibull survival curve determines the values of the discrete survival parameters in the CJS model, so the parameters in the likelihood are reduced from a potentially large set of discrete survival parameters to the small set of Weibull parameters that they map to. A summary of symbols used is presented in Table 3.

**Table 3:** Summary of symbols used (mostly from Ref. [13])

Symbol	Meaning
$S(t)$	Probability of survival beyond *t*, that is, Pr(lifetime ≥ *t*)
${R}_{i}(a)$	Number released of age *a* at occasion *i* (study data)
${m}_{ij}(a)$	Number of age *a*, when released at *i *are next caught at *j* (study data)
${p}_{i}$	Probability of capture at occasion *i*
${\phi }_{i}(a)$	Probability of surviving to the following day given alive at age *a*
${\nu }_{ij}(a)$	Probability of recapture at *j* when released at *i* at age *a*
${\chi }_{i}(a)$	Probability that those of age *a* are not re-caught again after occasion *i*
$\alpha ,\eta$	Shape and scale parameters of the Weibull distribution

Analysis uses the age-specific CJS likelihood equation [13, p. 74] written here as:

$$L \propto \mathop \prod \limits_{a} \mathop \prod \limits_{i = 1}^{T - 1} \left\{ {\chi_{i} \left( a \right)^{{R_{i} \left( a \right) - \sum m_{ij} \left( a \right)}} \mathop \prod \limits_{j = i + 1}^{T} \nu_{ij} \left( a \right)^{{m_{ij} \left( a \right)}} } \right\}$$

where ${R}_{i}\left(a\right)$ and ${m}_{ij}\left(a\right)$ are data arrays with examples given in the Data section, and for a mosquito of age *a* when released at occasion *i*, ${\nu }_{ij}\left(a\right)$ is the probability of next recapture at *j*, and ${\chi }_{i}\left(a\right)$ is the probability, of not being caught afterwards, so $\chi_{i} \left( a \right) = 1 - \mathop \sum \limits_{j} \nu_{ij} \left( a \right).$

This is a multinomial likelihood and, leaving age aside for the purposes of explanation, includes:

- the probability of no recapture (${\chi }_{i}$) raised to the power of the numbers not recaptured (${R}_{i}-\sum {m}_{ij}$); hence, the first term, and
- the probabilities of recapture (${\nu }_{ij}$) raised to the power of the number of recaptures (${m}_{ij}$); hence, the second term.

The parameters in the current analysis are relatively simple compared with the general form: *p* is the probability of capture on any recapture occasion, which is assumed time-independent, and $\underset{\_}{\phi }$ is a vector of probabilities, with element $\phi \left[k\right]$ the probability of surviving from age *k* to *k* + *1*.

Then,

$$\nu_{ij} \left( a \right) = \left( {1 - p} \right)^{j - i - 1} p \times \mathop \prod \limits_{k = i}^{j - 1} \phi \left[ {a + k - i} \right]$$

Conventionally, discrete survival probabilities (${\phi }_{k}$) are used in MR analyses (see Ref. [13]). The analysis for age-dependence when interest lies in discrete age classes is set out by Pollock et al. [21], as is common in some fields (e.g. birds: immature and mature). Analysis with many age classes requires many parameters and associated limits on precision. Parametric age-dependence on a continuum has been additionally utilised in this paper because it provides a more compact parameterisation and potentially increased precision. The parameter vector for an individual study under the discrete survival formulation (with a time-independent capture model) is $\left(p,{\phi }_{1},...{\phi }_{k}...\right)$, whereas under the compact parameterisation it is (for the Weibull survival model) $\underset{\_}{\theta }=\left(p,\alpha ,\eta \right)$.

A Weibull survival model is assumed with shape and scale parameters $\alpha$ and $\eta$. There are several textbook parameterisations of the Weibull, and the one adopted here corresponds to that coded in R. Note that the symbol for the Weibull scale parameter ($\sigma$) in the R parameterisation is replaced in this paper with $\eta$ because $\sigma$ is also commonly used for measures of dispersion. The Weibull distribution is fairly flexible though monotonic, and it includes the exponential as a special case when $\alpha =1$.

For the Weibull, the continuous survival function is:

$$S\left( t \right) = \exp \left( { - \left( {t/\eta } \right)^{\alpha } } \right)$$

The conditional survival over a time step is $S\left(k+1\right)/S\left(k\right)$ [22, p. 31], so the equation:

$$\phi \left( k \right) = \frac{{S\left( {k + 1} \right)}}{S\left( k \right)}$$

connects the continuous survival model with the discrete apparent survival of the CJS, in which $\phi \left(k\right)$ represents the probability of an animal alive at age $k$ surviving to $k+1$.

Weibull parameters are restricted to $\alpha >0$ and $\eta >0$. These constraints were implemented by numerical fitting with the Nelder–Mead method on the transformed variables $\text{log}\left(\alpha \right)$ and $\text{log}\left(\eta \right)$.

The EL of a mosquito is given by $\int S\left(t\right)\text{d}t$ and has an analytic solution for the Weibull model for known parameter values. To incorporate the parameter uncertainty in estimates of $\alpha$ and $\eta$, further analysis is required. The calculation in this paper of the variance of the conditional mean of the EL under a Weibull model is described in Additional File 1: Appendix S3.

Meta-analysis was carried out using the *metafor* package in R. The meta-analysis on expected lifetimes used a log transformation for this positive-value outcome, with inverse-variance weighting. The variance of the log-transformed EL was approximated using the ‘delta method’, that is:

$${\text{var}} \;\log \left( {{\text{EL}}} \right) \approx \left\{ {\frac{{d\,\log \left( {{\text{EL}}} \right)}}{{d\left( {{\text{EL}}} \right)}}} \right\}^{2} \cdot {\text{var}} \left( {{\text{EL}}} \right) = \frac{{{\text{var}} \left( {{\text{EL}}} \right)}}{{\left( {{\text{EL}}} \right)^{2} }}$$

The pooled estimate from the meta-analysis used a random-effects model to account for heterogeneity, which incorporates extra ‘between-study’ variation in the estimates.

Each study receives its own (constant) capture probability, which means there are as many capture parameters as studies; however, it is the survival parameters that are of primary interest and the capture parameters serve a modelling function only. In the analysis with genus as a moderator, there is an average for each group (e.g. for genus *Anopheles*) shared by those studies.

Three sensitivity analyses were carried out with alternative constraints:

1. For the overall model, with 0 < *p* < 0.05 and $\alpha \ge$ 1. The analysis asserts increasing or constant mortality with age, and rules out capture probabilities > 0.05, which may be implausible.
2. For the genus-specific model, 0 < *p* < 0.05 and $\alpha$ >0.1. The boundary on low values of $\alpha$ is a practical step to help avoid numerical difficulties, as discussed elsewhere.
3. For the genus-specific model, a meta-analysis excluding any studies with $\alpha <1$, where simulations showed estimation, produced a high root mean square error (RSME) (Additional File 1: Appendix S4).

### Simulations

Simulations of known-age MR experiments were carried out using known parameter values and known age, with time-independent capture probability and age-dependent survival. Four Weibull-derived survival models were used to generate simulated data, one exponential ($\alpha =1$), two with larger shoulders and increasing mortality with age ($\alpha >1$) and one where mortality fell with age ($\alpha <1$). These survival curves are shown in Additional File 1: Supplementary Fig. S2 along with their parameter values. The capture probability was set to 0.01 throughout, and there were 1000 runs in each scenario. When summarising scenarios, simulated data were trimmed where $\widehat{\alpha }$ > 30 or $\widehat{\eta }$> 30. The proportion of simulations with these outliers was 0.13.

In broad terms, the simulations showed that bias and variance reduces with more recapture occasions and larger cohort sizes, with younger release ages, and with more releases. The following inclusion criteria were adopted, when mosquitoes are released at known age, to give broadly accurate estimates (details below): releases at young age $\le 3$, a sufficient number of recapture occasions $\ge 8$ and

1. With single release, cohort size $R\ge 1000$
2. With multiple releases, $R\ge 500$

The heuristic reasoning for allowing smaller cohort sizes for multiple release experiments (500 versus 1000 for single release) is that the resulting loss of efficiency from the smaller cohorts is somewhat balanced by further releases made within the same experiment. Studies that did not meet the inclusion criteria were excluded from the meta-analysis (see Appendix S2).

The statistical performance of the main outcome of interest in the present study $\text{EL}$, along with results for $\alpha$ and $\eta$, is summarised in Appendix S4. Under the inclusion criteria, it can be seen that the bias of $\widehat{\text{EL}}$ is low (magnitude $\lesssim$ 0.5). Furthermore the RMSE of EL is rather smaller than the RMSE of $\alpha$ when $\alpha \gtrsim 2$ (scenarios a and b). However, when $\alpha <1$ (scenario d), the RMSE of $\widehat{\text{EL}}$ is large. The main conclusion of the simulations is that the bias of $\widehat{\text{EL}}$ may be reduced to low-moderate levels by the inclusion criteria but that the RMSE of $\widehat{\text{EL}}$ is especially high when $\alpha <1$. This is the region where Weibull variables are inherently most variable, and any estimates can be very imprecise.

## Results

Of the 30 selected studies, a further 3 were excluded where fitting experienced numerical difficulties (Supplementary Fig. S1), specifically that the variance–covariance matrix could not be estimated. The point estimates of $\alpha$ for these studies were very small (< 0.1). This led to a final set of 27 suitable MR experiments.

Some characteristics of the selected study datasets are presented in Table 2.

Some studies contained information from separate experiments, not all of which met the inclusion criteria. Also, the datasets may have more specific characteristics than the study as a whole: for example, the Yamar dataset used contained *Ae. vexans* neonates, whereas the study as a whole included *Cu. poicilipes*.

**Fig. 1:**
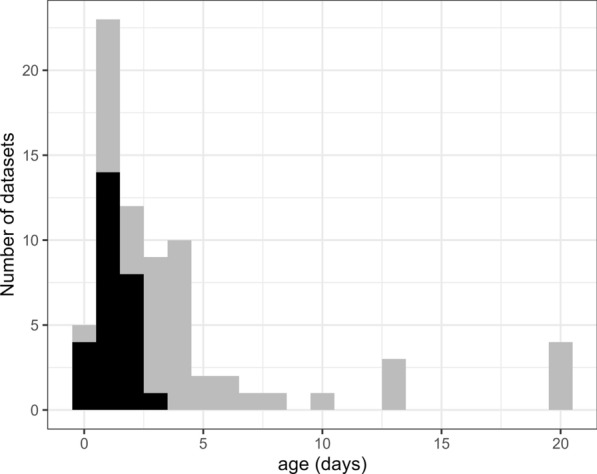
Ages released from all available known-age datasets (grey) and datasets used in this analysis after exclusions (black). For datasets with multiple ages, this is the age of the oldest cohort

A histogram of the oldest age of the cohorts at release of all available known-age studies, and those used in this study, is shown in Fig. 1. A large number of potentially informative studies were excluded under the criteria in place in the present study, foremost a restriction to younger-age releases.

**Fig. 2:**
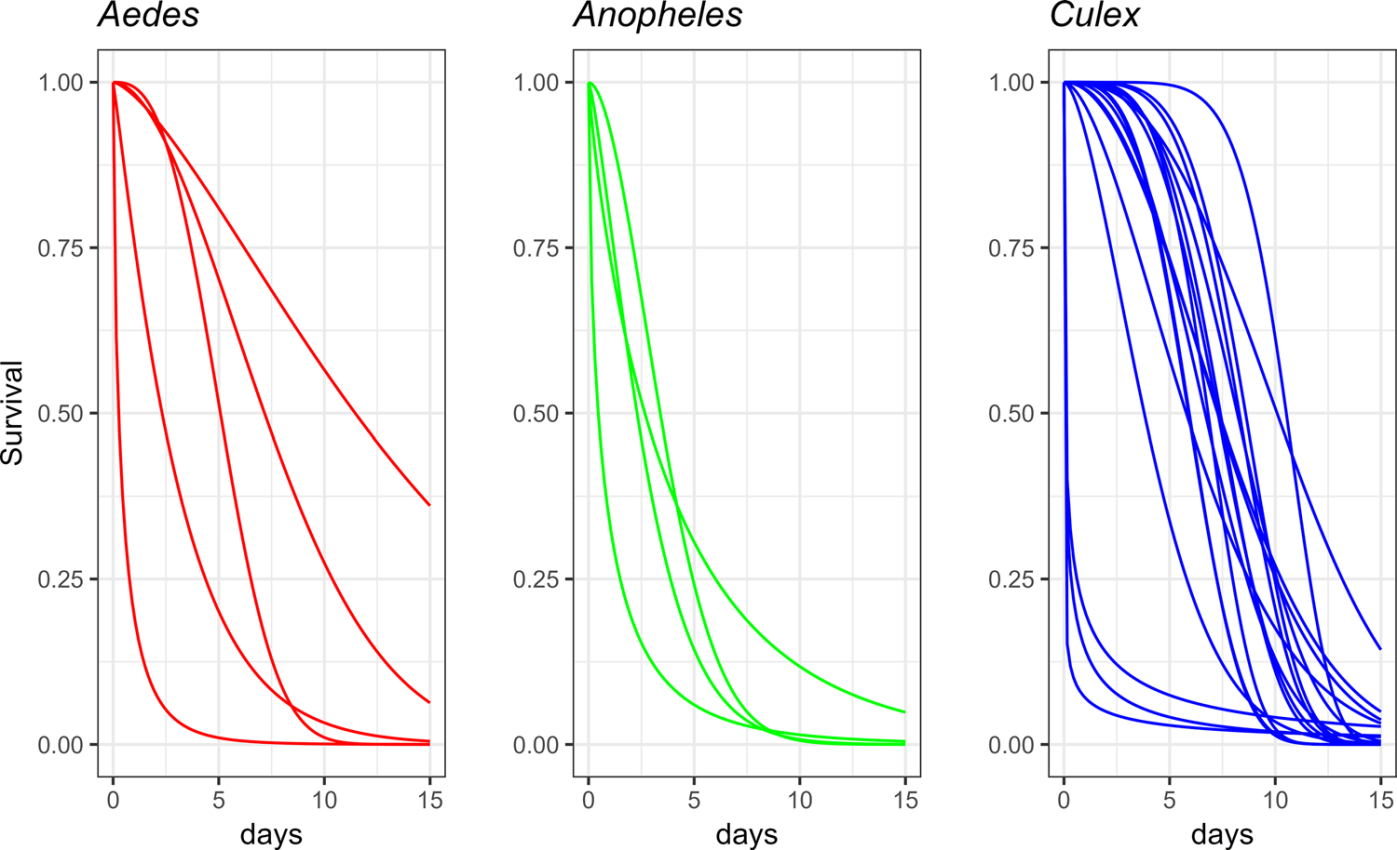
Weibull survival curves from parameter values estimated with MR studies, by genus

**Fig. 3:**
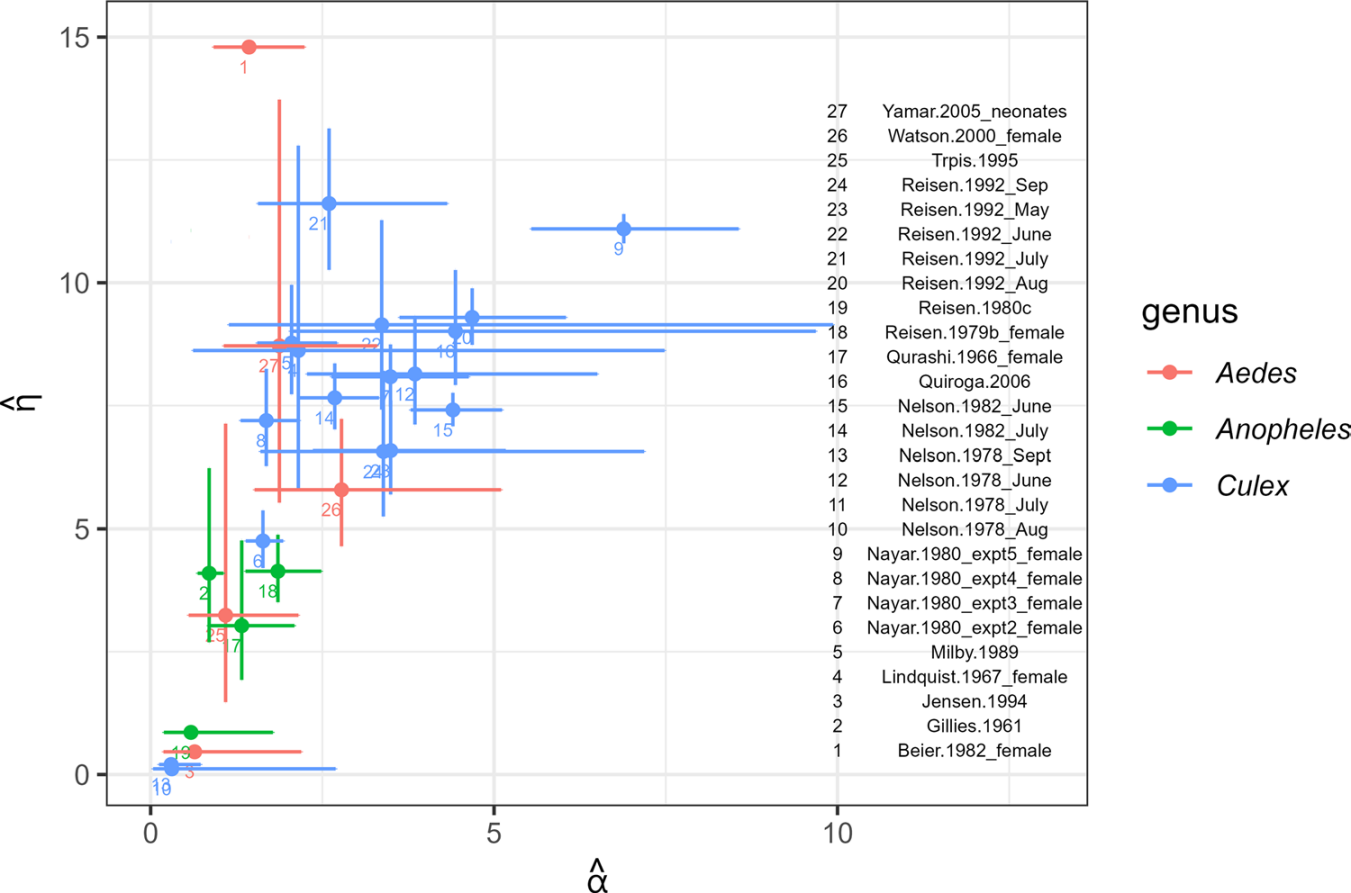
Parameter estimates of the Weibull model by study, with 95% confidence interval. $\alpha$ is the shape and $\eta$ the scale parameter of the Weibull distribution

The estimated survival curves are shown in Fig. 2. In those studies with a shouldered survival curve ($\alpha \gtrsim 1.5$), survival declines in older age, sometimes quickly, whereas mortality is high early on in those studies with $\alpha <1$. The results appear to show that the shouldered curves are associated with *Culex* mosquitoes. The studies and genera can be identified on the scatterplot in Fig. 3, which shows point estimates across the parameter space. There appear to be constellations of points by genus, with *Culex* at higher values of $\alpha$, and *Aedes* and *Anopheles* with smaller values often approaching 1. In the lower left-hand corner are estimates where $\alpha$ is small. These datasets are obtained from three studies [18, 23, 24]. The MR data for these datasets indicate strong peaks in early recapture, and have small EL with high variance. The simulation results (Appendix S4) showed high estimator variance (poor precision) for small $\alpha$.

**Fig. 4:**
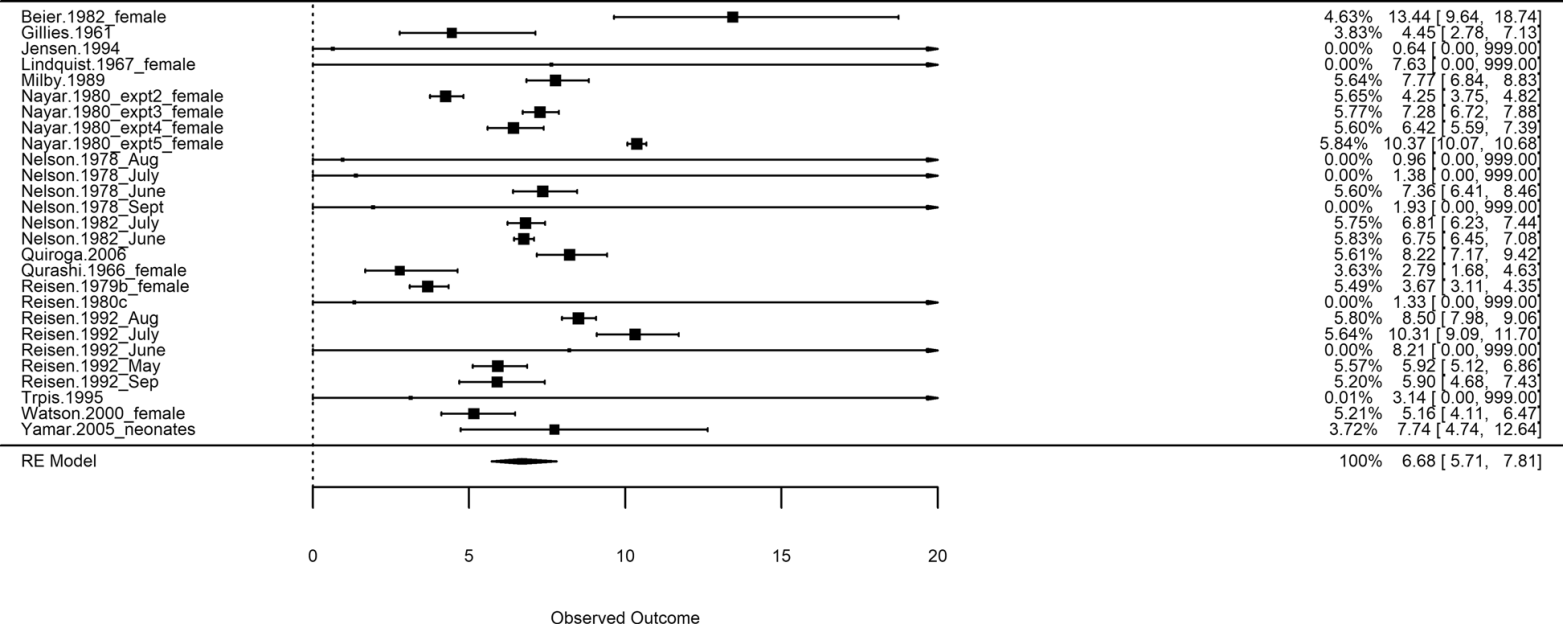
Forest plot of expected lifetimes from meta-analyses using an overall meta-analysis model with random effects. The pooled effect with 95% CLs is shown graphically as a diamond in the bottom entry. Note that studies with very large upper limits have had the upper limit truncated at an arbitrary large value (20) for presentation

A forest plot of the expected lifetimes is shown in Fig. 4 for the overall model (not separated by genus). As discussed, some studies provide very imprecise information and therefore carry negligible weight in the meta-analysis. The pooled estimate is shown as a diamond at the bottom of the figure. The average expected lifetime (*n* = 27) was 6.68 (95% CL 5.71–7.81) days. A summary is shown in Table 4. The sensitivity analyses of the overall model with the constraint $\alpha >1$, enforcing non-decreasing mortality, gave similar results with an EL of 6.76 (95% CL 5.86–7.81, *n* = 27).

**Table 4:** Model fitting results. See text for further explanation of sensitivity analyses 1-3. I^2^ is the heterogeneity statistic from the meta-analysis

Model	Survival	Sensitivity analysis	*I*^2^	Genus	*n*	EL
Overall	Weibull		97.0		27	6.68 (5.71–7.81)
Overall	Weibull	1	97.7		21	6.76 (5.86–7.81)
Overall	Exponential		96.9		30	4.69 (3.87–5.68)
By genus	Weibull		95.3	*Aedes*	5	7.92 (5.57–11.25)
				*Anopheles*	4	3.61 (2.51–5.18)
				*Culex*	18	7.22 (6.26–8.33)
By genus	Exponential		96.3	*Aedes*	6	3.84 (2.54–5.80)
				*Anopheles*	6	3.86 (2.51–5.95)
				*Culex*	18	5.33 (4.18–6.79)
By genus	Weibull	2	98.6	*Aedes*	5	7.03 (4.32–11.42)
				*Anopheles*	4	3.13 (1.91–5.13)
				*Culex*	18	6.16 (4.87–7.78)
By genus	Weibull	3	96.5	*Aedes*	4	7.92 (5.55–11.30)
				*Anopheles*	2	3.33 (2.17–5.12)
				*Culex*	15	7.22 (6.24–8.34)

For comparison, a meta-analysis using an age-independent model (*n* = 30) gave an EL of 4.69 (95% CL 3.87–5.68) days. The average of the probability of daily survival using the traditional regression approach (*n* = 30) was $\overline{p} = 0.805$, which equates to an EL of $- 1/{\text{log}}\left( {\overline{p}} \right) = 4.6\;{\text{days}}{.}$

**Fig. 5:**
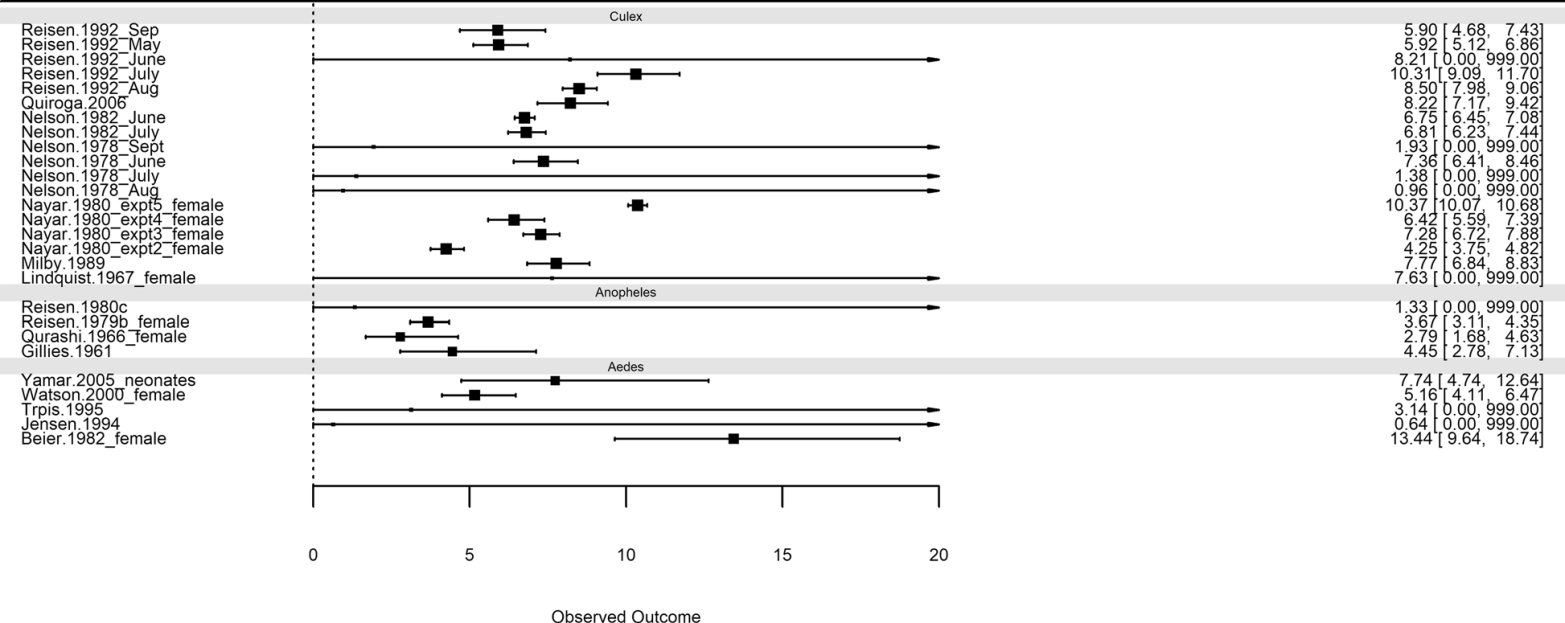
Forest plot of expected lifetimes from meta-analyses with genus included as a moderator. Note that studies with very large upper limits have had the upper limit truncated at an arbitrary large value (20) for presentation

The results when using genus as a moderator in the meta-analysis are presented in Table 4 and the forest plot shown in Fig. 5. The smaller point estimate of EL for *Anopheles* (3.61) is apparent in the plot and in the summary information compared with *Culex* (7.22) and *Aedes* (7.92).

The sensitivity analyses only meta-analysing studies with $\widehat{\alpha }\ge 1$ (Table 4) made little difference to the estimated values. The sensitivity analyses with bounds on $\alpha$ produced modest reductions in estimates, for example, the point estimate for EL for *Culex* changed from 7.22 to 6.16 with $\alpha >0.1$.

## Discussion

This study synthesises an important subset of historical mark–recapture studies in which mosquitoes were released at known age. The studies were screened by their characteristics and filtered for their likely validity in light of simulations made of estimator properties. Estimates were meta-analysed to produce pooled estimates of expected lifetime, taking into account differing study precision and heterogeneity.

A relatively flexible survival model (Weibull) was further incorporated to relax the conventional constant hazard (exponential) modelling approach in the mosquito literature. This also provides a more compact numerical summary than the set of discrete survival probabilities used in conventional time-dependent survival mark–recapture analyses, which can include many parameters. [25]. Of note, the traditional scalar summary of lifetime by the probability of survival through a single day is replaced with a less rigid shape-scale pair that can still be visualised (Fig. 3).

Two historically common approaches to MR are a regression on log-transformed recaptures, and the modification of it owing to Buonaccorsi et al. [12] applying nonlinear regression. In these cases, constant mortality (exponential survival) is assumed for a single-release MR experiment with time-independent recapture probability. The CJS analysis in this study can provide similar analysis under the time-independent/exponential assumption (see Additional File 1: Appendix S1) but can also extend to age-varying capture probability and multiple release experiments and can in principle accommodate time-varying capture probability. It also explicitly models all recapture occasions, not just those up to the last observed recapture.

The EL results for *Anopheles* in this study was 3.61 (2.51–5.18) days (Table 4). In a recent synthesis [10] of the probability of daily survival among Anopheline mosquitoes, a pooled average of 0.73 was calculated for mark–recapture studies, corresponding to a similar EL of 3.2 days. The present analysis used a much smaller collection of studies, being restricted to a subset (known age, etc.) of mark–recapture studies. Nevertheless, the result here is likely much more reliable, primarily because studies with potentially poor estimates (e.g. insufficient releases), or uncontrolled ages on release, were excluded; information on uncertainty of estimates is incorporated into the meta-analysis (this was largely unavailable to Matthews et al. [10] working with extracted summary information); and because a more flexible model, allowing for age-dependence, was used. These results improve upon less formal selection and reporting of mosquito longevity e.g. [8] based their modelling on study-specific estimates ranging from 3.6 to 20 days.

There are differences in EL between mosquitoes in the three genera. Compared with the results for *Anopheles* just mentioned, higher estimates of EL were made for *Aedes* 7.92 (5.57–11.2) and for *Culex* 7.22 (6.26–8.33) (Table 4).

In interpreting these genus-specific results, the concept of ‘apparent survival’ (vis a vis survival) is important (e.g. McRea p. 58). What is estimated is the probability of surviving by not dying and not emigrating from the study area. This means that the estimated survival curves can relate to aspects of study design that influence dispersal, such as the spatial configuration and coverage of the recapture apparatus, the terrain or the use of baits (human or animal). For example, the study site used by Nelson et al. [23] was a ravine leading to a reservoir, which likely influenced the dispersal of the marked mosquitoes. The characterisation of the spatial environment of each study can be important but its complete influence on the data is beyond the scope of this paper. We wholly concur with the authors of Guerra et al. [9] that the ‘distribution, density and frequency of operation of sampling assets critically influenced recapture success’. The present study included restrictions on small studies in terms of release numbers and recapture attempts but did not include spatial distribution. The influence of emigration, which relates strongly to both study area and the spatial arrangement of recapture to release points, remains unexplored.

Other limitations of this study include the following: Mosquito populations in peopled areas are subject to interventions such as nets or drainage at both domestic and governmental levels. Our study excluded scientific interventions as reported in the titles of the publications, but this may only eliminate some more unusual (e.g. genetic manipulations or bacterial infections) and more intense (e.g. larvicide) experimental conditions—the distinction between pristine and manipulated populations remains blurred. Mosquitoes in mark–recapture studies are released in large batches, often of similar genetic provenance, raised in identical conditions, etc. As a result, the experimental variation between individuals is less than would be found were animals in reality randomly sampled in the wild. Goodness-of-fit tests were avoided in this study because of this non-independence. A modelling assumption made here is that within each study the probability of detection is time-independent. In most included studies, researchers usually used one or two recapture methods only, and this was maintained across the study. However, there were exceptions to this, e.g. Ref. [18] reports five different methods of recapture, and Lowe et al. [26] reports the use of human bait in the first hour after release only (though unstated, that appeared to be for the purpose of removing weakened individuals). The process of releasing emergent mosquitoes typically leads to release cohorts treated as homogenous age, but this can be a simplification, e.g. the authors of Eldridge et al. [16] defined age cohorts on the basis of the time between ‘peak emergence’ and release date.

A more serious weakness of the time-independent capture aspect of the model is that there can be low recapture rates in the early period, though this is not observed consistently and clearly related to the study-specific recapture technique and gonotrophic status. Assessing their recaptures, Kramer et al. [27] says ‘recapture rates on day 1 were low because females had not matured sufficiently to initiate host-seeking activity’ (see also a similar comment by Milby and Reisen [11]). The phenomenon can be seen, for example, in the study with the highest estimated value of $\alpha$ (furthest right in Figs. 2 and 3) from Nayar et al. [28], experiment 5, which has a sustained recapture rate over more than a week but also a delayed peak:

**Table:** 

2	3	4	5	6	7	8	9	10	11	12	13
119	213	306	203	205	106	124	118	102	38	6	0

Regarding this, the authors of Nayar et al. [28] commented that ‘mosquitoes when released tend to disperse appetentially and only after their first appetential needs are met do they settle down in their ecological habitats’. A reviewer also commented on cyclical recapture probabilities tracking blood meal digestion. There is clearly scope to improve on the time-independent capture part of the model.

Of the multitude of factors influencing the survival curves, this study has accounted for many. First, aspects of mark–recapture experimental design (single or multiple release occasions, single or multiple ages at release and irregular or regular recapture) can all be modelled by the CJS. Any individual experiment may hold specific capture characteristics, which will depend on the type of recapture apparatus and may depend on the marking procedure. Again, this is accounted for since each experiment has its own estimated capture parameter. These capture parameters are not of primary interest and are not taken to the stage of interpretation, but their influence on the data is modelled. Finally, the data are grouped by mosquito genus, as there are likely to be differences in (apparent) survival between these.

There remains considerable variation in parameter estimates as can be seen over the parameter space (Fig. 3) and in the estimated Weibull curves (Fig. 2). The heterogeneity estimate from the meta-analysis was very high (${I}^{2}=$ 97%) even after inclusion of genus in the meta-analysis (${I}^{2}$ = 95.3%). Factors varying across studies that contribute to ‘unexplained’ variation include biological characteristics such as species, aspects of the study site and external factors such as humidity and temperature. Note that where study design characteristics such as study area or use of bait may influence apparent survival, they can contribute to between-study differences/heterogeneity. At least some of this may be amenable to an extended future analysis, though this would likely be quite involved, e.g. imputation of humidity.

As is typical in reviews of heterogeneous material, some potentially subjective decisions are required. One difficulty is the definition of a MR experiment. A sequence of releases may be approached as a set of single-release experiments, or a single multiple-release experiment, and the decision may depend on the spatial and temporal separation of those releases, the preferred method of analysis and the way data are reported. In this paper, we intended to treat any set of releases in the same locality and overlapping in time that are distinguished by their marks as a single multiple-release experiment (e.g. Ref. [16]). However, the reporting of the data is the determining factor: for example, the authors of Reisen et al. [17] reported recaptures by the month of the experimental period. Brief details of the way that the included studies reported MR information are presented in Table 2.

To be included here, a study had to belong to the intersection of several criteria: meeting the design inclusion criteria including sufficient numbers released; age known at release; and appropriate recapture information supplied, preferably as m-array. With respect to the last of these, many potentially valuable studies were omitted from the present study owing to aspects of reporting. For example, Haramis and Foster [29] released four cohorts of mosquitoes at known age in large numbers (average cohort size about 1400) but did not report an m-array nor was the recapture by time information available for individual cohorts presented in a table or graph. Large releases of mosquitoes and summary recapture information were available from plots in Walker et al. [30], but the age of the mosquitoes at release, though known, was not clearly stated. The study by Abdel-Malek [31] used multiple releases at known age but reported results that were aggregated over the release dates.

The Weibull survival model used here is much less restrictive than the exponential; indeed, the exponential is a special case of it when $\alpha =1$. Still, there are plausible survival models other than the Weibull that may provide superior fits: Gompertz, Gompertz-Makeham, logistic, logistic-Makeham [3], generalised additive models [5] and a quadratic hazard function [4]. These or other alternatives have not been explored here. There were numerical difficulties in our analysis in a few datasets at small values of $\widehat{\alpha }$, and other survival models may prove more successful. It is worth mentioning that the monotonicity of the Weibull curve may prove overly stiff. There has been limited evidence of a mortality inflexion [4], and the authors of Styer et al. [3] report a decline at very advanced laboratory ages; see their Fig. 1).

The survival curve of a vector relates to disease epidemiology via the expected infectious lifetime (EIL), which is the lifetime of a mosquito after the extrinsic incubation period (EIP) of the parasite, i.e. expected lifetime of the mosquito once it becomes infectious. The relevant equation for EIL is given by Klein and Moeschberger [22], here written ${\int }_{\text{EIP}}^{\infty }S\left(t\right)\cdot \text{dt}/S\left(\text{EIP}\right)$, and could be calculated for any survival curve presented in this paper. However EIP is an elaborate concept that varies by parasite and other biotic factors [32]; therefore, it has not been explored further here.

In the present work, studies were analysed independently, and the main summary outcomes of expected longevity were subsequently pooled with a standard meta-analysis. The inclusion criteria in the present study specified large cohorts of at least 500, a minimum number of recapture occasions (eight) and younger ages at release ($\le 3$), so 43 datasets were excluded (Supplementary Fig. S1). Despite the large body of research on these genera, there were ultimately few selected datasets particularly for *Anopheles* (*n* = 4) and *Aedes* (*n* = 5). There is consequently an increased possibility of insufficient samples across what is a heterogeneous space, and potential overfitting.

A superior analysis approach could be to use an integrated (Bayesian) analysis in which study parameters are drawn from a prior distribution, so the studies can share information. Studies which were rejected on an individual basis on considerations such as the number of releases might then be used. One particular problem that limited the inclusion of studies in the current analysis was that experiments with mosquitoes released at known older ages were rejected (Fig. 1), despite the inherent value of empirical information at older ages towards the tail of the survival curve. For example, a valuable series of datasets by Maciel-de-Freitas and co-authors (e.g. Ref. [33]) were excluded here because they were carried out with mosquitoes of older age. In isolation, these studies cannot reliably inform the whole survival curve and give potentially inaccurate estimates under simulation, but within an integrated analysis they could contribute. A final benefit of an integrated analysis over the approach of the present work is that some of the numerical difficulties encountered near parameter boundaries, especially small values of $\alpha$, might be avoided.

## Conclusions

This study synthesised known-age MR mosquito studies whilst also restricting to those with certain favourable characteristics (on the basis of numbers released and recaptured and their ages at release). An age-dependent Weibull survival model was assumed rather than the often-used age-independent (exponential) model. The estimated EL for female *Aedes* and *Culex* is about a week, but for *Anopheles*, it is about 4 days. The estimated quantities for *Aedes* and *Anopheles* require a caveat that the number of datasets, after exclusions, is small (*n* = 5 and *n* = 4, respectively). Heterogeneity of EL across the MR studies is high and, among the factors which potentially affect estimates of EL, the spatial configuration, including study area, of the recapture experiments is emphasised. A future integrated (Bayesian) analysis could offer an important advantage by using further studies (those with fewer releases and recaptures and older release cohorts), and this larger assembly could allow for further adjustment for study characteristics.

## Supplementary Information

**Additional file 1. Text Appendix S1.** Similarity of CJS and regression estimates. **Text Appendix S2.** Study selection. **Fig. S1.** Study and dataset selection flowchart. **Text Appendix S3.** Variance of Weibull expected lifetime when parameters are uncertain. **Text Appendix S4.** Statistical performance of estimators of EL. **Fig. S2.** Four Weibull survival curves and their shape ($\alpha$) and scale ($\eta$) parameter values in parentheses. **Fig. S3.** The theoretical partial derivative of EL with respect to parameters $\alpha$ and $\eta$. **Tables S1 and S2**. Statistical performance of estimators.

**Additional file 2.** File containing a list of references that provided data for analysis, and a list of further mark–recapture studies that were assessed, not present in [99].

**Additional file 3.** File containing data and R code.

## Data Availability

The datasets used in the current study are available in the supplementary material, along with code to generate the main meta-analysis results.
