# Supplementary material for: Mosquito survival from mark–recapture studies releasing at known age
Source: Parasit Vectors. 2025 Nov 10;18:455. doi: 10.1186/s13071-025-07024-2 (PMC12604168; doi:10.1186/s13071-025-07024-2)
Supplement: Supplementary file 1 — Additional file 1. Text Appendix S1. Similarity of CJS and regression estimates. Text Appendix S2. Study selection. Fig. S1. Study and dataset selection flowchart. Text Appendix S3. Variance of Weibull expected lifetime when parameters are uncertain. Text Appendix S4. Statistical performance of estimators of EL. Fig. S2. Four Weibull survival curves and their shape ($\alpha$α) and scale ($\eta$η) parameter values in parentheses. Fig. S3. The theoretical partial derivative of EL with respect to parameters $\alpha$α and $\eta$η. Tables S1 and S2. Statistical performance of estimators. [file 13071_2025_7024_MOESM1_ESM.pdf]

Appendices

S1 Similarity of CJS and Regression Estimates

The CJS model with time-independent capture was defined in the main text with the following probabilities for individuals released at occasion i, of age a, and recaptured at j:

$$v_{ij}(a) = p(1-p)^{j-i-1} \prod_{k=i}^{j-1} \phi\,[a+k-i]$$

for i=1,2,… and i<j.

With survival now assumed time-independent and for a single release experiment with releases from occasion 1, then this probability reduces to:

$$v_{1j} = \phi^{j-1}(1-p)^{j-2}p$$

With R released at occasion 1, the expected number caught at recapture occasion j=2,… is :

$$E\left[m_j\right] = R\phi^{j-1}(1-p)^{j-2}p$$

or releasing at time 0 not 1:

$$= R\phi^{j}(1-p)^{j-1}p$$

This is the same expectation as that used more conventionally in the mosquito literature. For a set of observed counts of recaptures y_j, this can be solved by taking logarithms and carrying out a linear regression. Alternatively, in an improved approach, [12] solve by nonlinear least squares i.e. minimising $\sum\left(E\left(y_j\right) - y_j\right)^2$.

S2 Study selection

The process of selecting datasets from mosquito MR studies of known age is show in fig. S1.

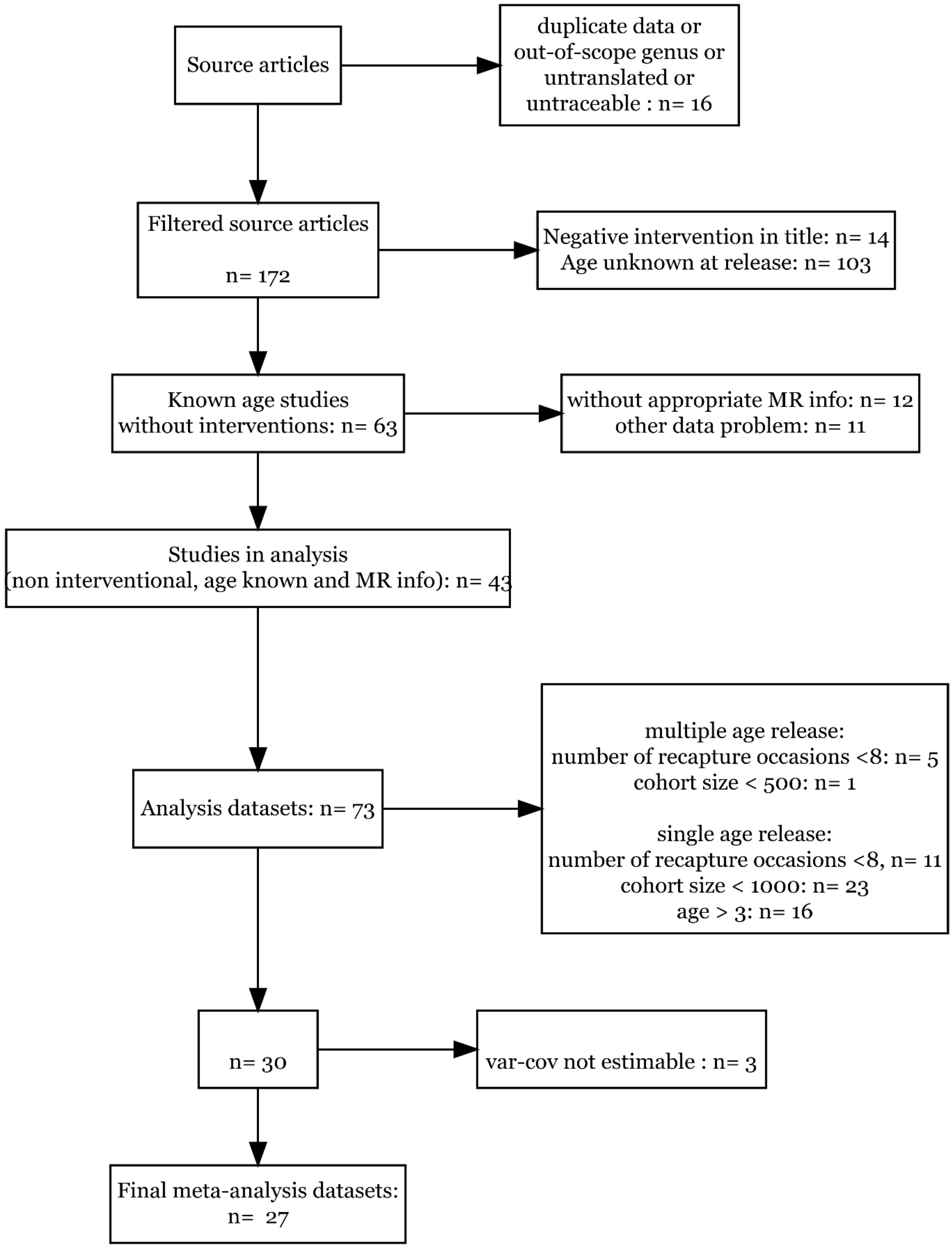

Figure S1. Study and dataset selection flowchart. Abbreviations: MR, mark-recapture; EL, expected lifetime.

S3 Variance Of Weibull Expected Lifetime When Parameters Are Uncertain

Let X represent the lifetime of a mosquito. We suppose that the lifetime X has Weibull pdf $f(X|\alpha,\eta) = (\alpha/\eta)(x/\eta)^{\alpha-1}\exp(-(x/\eta)^{\alpha})$. An analytic expressions is available for the expected lifetime $E[X] = \eta\Gamma(1 + 1/\alpha)$, where $\Gamma(\quad)$ is the mathematical gamma function.

This expression makes the assumption that the parameters (α,η) are known; further analysis is required to extend the uncertainty, as follows.

From a fitting process for each study we obtain estimates $\hat{\alpha}$ and $\hat{\eta}$ and associated covariance matrix, which we treat as random variables with pdf $BVN(\mu,\Sigma)$ where BVN() denotes the bivariate normal, centred on the mean $\mu = (\bar{\alpha},\bar{\eta})$ with covariance matrix Σ.

For the uncertainty in expected Weibull lifetime X given variation in parameters α and η, the variance of the conditional mean is

$$\int_{-\infty}^{\infty}\int_{-\infty}^{\infty}(E[X(\alpha,\eta)] - E[X(\bar{\alpha},\bar{\eta})])^2\, BVN(\mu,\Sigma)d\alpha.d\eta$$

The Weibull distribution has positive parameters ($\alpha > 0$ and $\eta > 0$) as explained in the main text. We use a log() transformation to allow estimation of the BVN over an unbounded plane, so that $\alpha' = log(\alpha)$ and $\eta' = log(\eta)$. The form of the equation is then

$$\int_{-\infty}^{\infty}\int_{-\infty}^{\infty}\left(E\left[X\left(exp(\alpha'),exp(\eta')\right)\right] - E\left[X\left(exp(\bar{\alpha}'),exp(\bar{\eta}')\right)\right]\right)^2 BVN(\mu',\Sigma')d\alpha'.d\eta'$$

where μ' and Σ' is the mean and covariance matrix of the parameters α' and η'.

The mean values are known analytically but the variance of the conditional mean required computation. There were numerical difficulties under this computation for values of $\alpha \lesssim .3$. These arise because the gamma function within the analytic equation reaches very high values as its argument increases.

To deal with this, a lower bound on α of 0.1 was used for this calculation, with a corresponding $E[X] = \eta\Gamma(1 + 1/\ 0.1\ ) = \eta \times 3628800$. Under this procedure the integration remains within machine precision, while the estimates of conditional variance remain high (and therefore these studies retain negligible weight in the meta-analysis). A further practical step was to use finite integration limits, specifically quantiles corresponding to small probabilities (p=1e-15).

S4 Statistical performance of estimators of EL

Four survival scenarios were investigated as shown in fig. S2. Results for estimates of α, η and EL are shown in this section, using the inclusion criteria developed and described in the Simulations section. Bias, variance and root-mean-square-error (RMSE) were measured. Single release scenario results are shown in Table S1 and multiple release in Table S2 below.

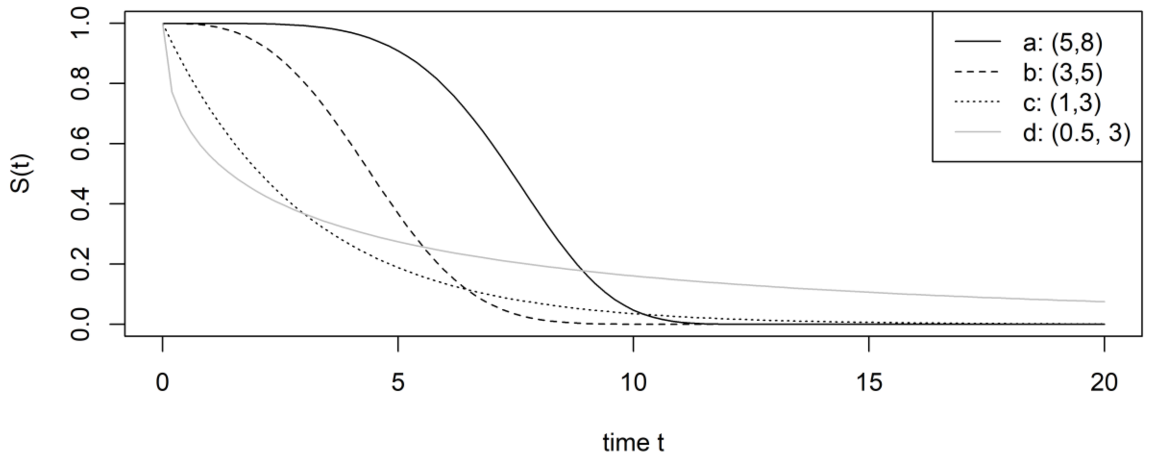

Figure S2: Four Weibull survival curves and their shape (α) and scale (η) parameter values in parentheses. Estimates of these were made in simulations. Curve c with $\alpha = 1$ is also an exponential curve.

The tables show that for these inclusion criteria, levels of bias are low (magnitude usually $\lesssim 1$) for α, η and EL in scenarios a-c. Even for the worst-performing estimator (of parameter α) in the worst-performing scenario (d), the magnitude of bias is usually $\lesssim 2$.

Though there is a moderate to high magnitude (>3 or much larger) in the variance and RMSE of $\hat{\alpha}$ when $\alpha > 1$ (scenarios a-b), the variance and RMSE of $\widehat{EL}$ is of much lower magnitude, especially in scenario b. This may be explained by the insensitivity of EL to α when $\alpha \gtrsim 2$, as shown by the partial derivatives in fig. S3. However the variance and RMSE of $\hat{\alpha}$ and $\widehat{EL}$ remain substantial for $\alpha < 1$ (scenario d), when there is a strong dependence of EL on α (fig. S2).

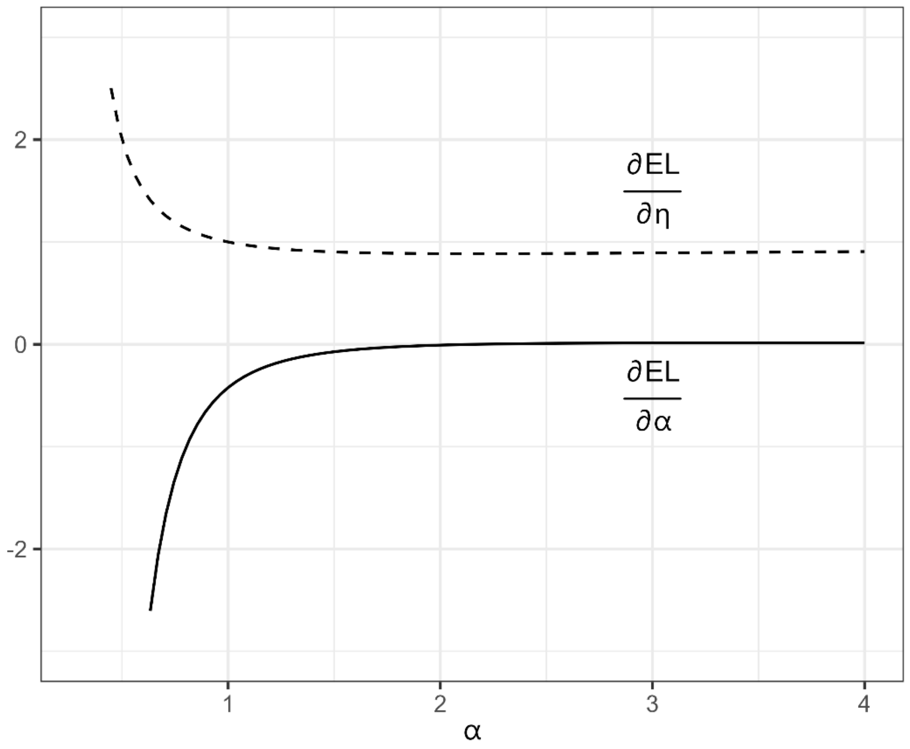

Figure S3: The theoretical partial derivative of EL with respect to parameters α and η. For values of α above around 2, the EL is linearly related to η and almost independent of α. However for $\alpha < 1$ EL quickly becomes sensitive to both.

Estimator properties by simulation with single release at age 1, R=1000, and 8 recapture occasions.

scenario	EL	mean (EL)	var (EL)	bias (EL)	RMSE (EL)	alpha	mean (alpha)	var (alpha)	bias (alpha)	RMSE (alpha)	eta	mean (eta)	var (eta)	bias (eta)	RMSE (eta)
a	7.35	7.16	8.17	-0.19	2.86	5.00	5.37	22.88	0.37	4.80	8.00	7.19	6.18	-0.81	2.61
b	4.46	4.48	0.86	0.01	0.93	3.00	4.42	12.20	1.42	3.77	5.00	4.89	0.83	-0.11	0.92
c	3.00	3.14	4.63	0.14	2.16	1.00	1.96	10.40	0.96	3.37	3.00	2.87	5.17	-0.13	2.28
d	6.00	6.34	23.20	0.34	4.83	0.50	2.53	19.80	2.03	4.89	3.00	4.21	16.66	1.21	4.26

Table S1: Estimator properties with single release.

Estimator properties by simulation with multiple release at age 1, R=500, and 8 recapture occasions.

scenario	ntr	EL	mean (EL)	var (EL)	bias (EL)	RMSE (EL)	alpha	mean (alpha)	var (alpha)	bias (alpha)	RMSE (alpha)	eta	mean (eta)	var (eta)	bias (eta)	RMSE (eta)
a	3	7.35	6.83	13.93	-0.52	3.77	5.00	5.05	27.31	0.05	5.23	8.00	6.85	10.57	-1.15	3.45
a	5	7.35	6.67	11.88	-0.68	3.51	5.00	5.29	29.03	0.29	5.40	8.00	6.94	9.89	-1.06	3.32
b	3	4.46	4.46	0.52	-0.01	0.72	3.00	4.21	10.84	1.21	3.51	5.00	4.89	0.66	-0.11	0.82
b	5	4.46	4.45	0.31	-0.01	0.56	3.00	3.74	6.00	0.74	2.56	5.00	4.92	0.37	-0.08	0.62
c	3	3.00	3.12	4.29	0.12	2.07	1.00	1.78	5.54	0.78	2.48	3.00	2.91	5.19	-0.09	2.28
c	5	3.00	2.95	2.66	-0.05	1.63	1.00	1.39	3.36	0.39	1.87	3.00	2.65	3.81	-0.35	1.98
d	3	6.00	6.34	21.09	0.34	4.60	0.50	2.20	15.35	1.70	4.27	3.00	3.95	13.54	0.95	3.80
d	5	6.00	6.34	20.98	0.34	4.59	0.50	1.75	8.22	1.25	3.13	3.00	3.96	13.43	0.96	3.79

Table S2: Estimator properties with multiple release.
