# Supplementary material for: Mosquito survival from mark–recapture studies releasing at known age
Source: Parasit Vectors. 2025 Nov 10;18:455. doi: 10.1186/s13071-025-07024-2 (PMC12604168; doi:10.1186/s13071-025-07024-2)
Supplement: Supplementary file 2 — Additional file 2. File containing a list of references that provided data for analysis, and a list of further mark–recapture studies that were assessed, not present in [99]. [file 13071_2025_7024_MOESM2_ESM.pdf]

Supplementary File: Additional References

List of selected references containing data for use in analysis.

1. Beier JC, Berry W, Craig G. Horizontal distribution of adult *Aedes triseriatus* (Diptera: Culicidae) in relation to habitat structure, oviposition and other mosquito species. J Med Entomol. 1982;19(3):239–47.

2. Elizondo-Quiroga E, Flores-Suarez A, Elizondo-Quiroga D, et al. Gonotrophic cycle and survivorship of *Culex quinquefasciatus* using sticky ovitraps in Monterrey, northeastern Mexico. J Am Mosq Control. 2006;22:10–4.

3. Gillies MT. Studies on the dispersion and survival of *Anopheles gambiae* Giles in East Africa, by means of marking and release experiments. B Entomol Res. 1961;52(1):99–127.

4. Jensen T, Washino R. An assessment of the biological capacity of a Sacramento Valley population of *Aedes melanimon* to vector arboviruses. Am J Trop Med Hyg. 1991;44:355–63.

5. Jensen T, Washino R. Comparison of recapture patterns of marked and released *Aedes vexans* and *Ae. melanimon* (Diptera: Culicidae) in the Sacramento Valley of California. J Med Entomol. 1994;31(4):607–10.

6. Lindquist A, Ikeshoji T, Grab B, de Meillon B, Khan Z. Dispersion studies of *Culex pipiens fatigans* tagged with 32P in the Kemmendine area of Rangoon, Burma. Bull WHO. 1967;36(1):21–37.

7. Liu QY, Liu XB, Zhou GC, Jiang JY, Guo YH, Ren DS, et al. Dispersal range of *Anopheles sinensis* in Yongcheng City, China by mark-release-recapture methods. PLoS One. 2012 Nov;7(11):8.

8. Midega JT, Mbogo CM, Mwnambi H, Wilson MD, Ojwang G, Mwangangi JM, et al. Estimating dispersal and survival of *Anopheles gambiae* and *Anopheles funestus* along the Kenyan coast by using mark-release-recapture methods. J Med Entomol. 2007 Nov;44(6):923–9.

9. Milby M, Reisen W. Estimation of vectorial capacity: vector survivorship. Bull Soc Vector Ecol. 1989;14(1):47–54.

10. Nayar J, Provost M, Hansen C. Quantitative bionomics of *Culex nigripalpus* (Diptera: Culicidae) populations in Florida 2. distribution, dispersal and survival patterns. J Med Entomol. 1980;17:40–50.

11. Nelson R, Milby M. Autogeny and blood-feeding by *Culex tarsalis* (Diptera: Culicidae) and the interval between oviposition and feeding. Canadian Entomologist. 114:515–21.

12. Nelson R, Milby M, Reeves W, Fine P. Estimates of survival, population size, and

emergence of *Culex tarsalis* at an isolated site. Annals of the Entomological Society of America. 1978;71:801–8.

13. Qurashi M, Faghih M, Esghi N. Flight range, lengths of gonotrophic cycles, and longevity of P32-labeled *Anopheles stephensi mysorensis*. Journal of Economic Entomology. 1966;59(1):50–5.

14. Reisen W, Aslamkhan M. A release-recapture experiment with the malaria vector, *Anopheles stephensi*, Liston with observations on dispersal, survivorship, population size, gonotrophic rhythm and mating behaviour. Ann Trop Med Parasit. 1979;73:251–69.

15. Reisen W, Mahmood F, Parveen T. *Anopheles culicifacies* Giles: a release-recapture experiment with cohorts of known age with implications for malaria epidemiology and genetical control in Pakistan. Trans R Soc Trop Med Hyg. 1980;74:307–17.

16. Reisen W, Milby M, Meyer R. Population dynamics of adult Culex mosquitoes (Diptera: Culicidae) along the Kern River, Kern County, California, in 1990. J Med Entomol. 1992;29(3):531–43.

17. Trpis M, Hausermann W, Craig G. Estimates of population size, dispersal, and longevity of domestic *Aedes aegypti aegypti* (Diptera: Culicidae) by mark-release-recapture in the Village of Shauri Moyo in Eastern Kenya. J Med Entomol. 1995;32:27–33.

18. Watson T, Saul A, Kay B. *Aedes notoscriptus* (Diptera: Culicidae) survival and dispersal estimated by mark-release-recapture in Brisbane, Queensland, Australia. J Med Entomol. 2000;37(3):380–4.

19. Yamar B, Diallo D, Kebe C, Diallo M. Aspects of bioecology of two Rift Valley fever virus vectors in Senegal (West Africa): *Aedes vexans* and *Culex poicilipes* (Diptera: Culicidae). J Med Entomol. 2005;42:739–50.

Mosquito mark-recapture references additional to those found by Guerra et al. (2014) that were assessed.

1. Villela D, Garcia G, Maciel-de-Freitas R. Novel inference models for estimation of abundance, survivorship and recruitment in mosquito populatoins using mark-release-recapture data. PLoS Negleced Tropical Diseases. 2017;11(6).

2. Villela D, Codeço C, Figueiredo F, et al. A Bayesian Hierarchical Model for Estimation of Abundance and Spatial Density of Aedes aegypti. PLoS One. 2015;10(4).

3. Tsuda Y, Hamezaki H. Mark-release-recapture study on movement of mosquitoes: individual marking method and short-term study of *Aedes albopictus* and *Armigeres subalbatus* in residential area on Ishigaki island, Japan. Med Entomol Zool. 2014;65(2):61–6.

4. Trpis M, et al. Estimates of Population Size, Dispersal, and Longevity of Domestic *Aedes aegypti* (Diptera: Culicidae) by Mark—Release—Recapture in the Village of Shauri Moyo in Eastern Kenya. J Med Entomol. 1995;32:27–33.

5. Sheppard P, Macdonald W, Tonn R, Grabb B. The dynamics of an adult population of *Aedes aegypti* in relation to dengue haemorrhagic fever in Bangkok. J Animal Ecology. 1969;38(3):661–702.

6. Saul A. Estimation of survival rates and population size from mark-recapture experiments of bait-caught haematophagous insects. B Entomol Res. 1987;77(4):589–602.

7. Russell RC, Rao T. Observations on the longevity of *Anopheles culicifacies* imagines. Am J Trop Med Hygiene. 1942;22:517–33.

8. Ritchie SA, Montgomery B, Hoffman A. Novel estimates of *Aedes aegypti* population size and adult survival based on Wolbachia releases. J Med Entomol. 2013;50(3):624–31.

9. Reisen W, Aslamkhan M. A release-recapture experiment with the malaria vector, *Anopheles stephensi*, Liston with observations on dispersal, survivorship, population size, gonotrophic rhythm and mating behaviour. Ann Trop Med Parasit. 1979;73:251–69.

10. Qurashi M, Faghih M, Esghi N. Flight Range, Lengths of Gonotrophic Cycles, and Longevity of P32-Labeled *Anopheles stephensi mysorensis.* Journal of Economic Entomology. 1966;59(1):50–5.

11. McLelland, et al. 1973 Results of preliminary mark-release-recapture trials with *Aedes nigromaculis*. Proc 41st Ann. Conf. California Mosq. Control Association. pp107-8.

12. Lowe R, Schreck C, Hobbs J, Dame D, Lofgren C. Studies on the flight range and survival of *Anopheles albimanus* Wiedemann in El Salvador II: Comparisons of release methods with sterile and normal adults in wet and dry seasons. Mosquito News. 35(2):160–8.

13. Liu QY, Liu XB, Zhou GC, Jiang JY, Guo YH, Ren DS, et al. Dispersal Range of *Anopheles sinensis* in Yongcheng City, China by Mark-Release-Recapture Methods. PLoS One. 2012 Nov;7(11):8.

14. Linthicum K, Bailey C. Observations on the dispersal and survival of a population of *Aedes lineatopennis* (Ludlow) (Diptera: Culicidae) in Kenya. B Entomol Res. 1985;75:661–70.

15. Lindquist A, Ikeshoji T, Grab B, de Meillon B, Khan Z. Dispersion studies of *Culex pipiens* fatigans tagged with 32P in the Kemmendine area of Rangoon, Burma. Bull WHO. 1967;36(1):21–37.

16. Hobbs J, Lowe R, Schrek C. Studies of flight range and survival of *Anopheles albimanus* Wiedemann in El Salvador: I Dispersal and survival during the dry season. Mosquito News. 1974;34:389.

17. Epopa PS, Millogo AA, Collins CM, North A, Tripet F, Benedict MQ, et al. The use of sequential mark-release-recapture experiments to estimate population size, survival and dispersal of male mosquitoes of the *Anopheles gambiae* complex in Bana, a west African humid savannah village. Parasites & Vectors. 2017 10:376.

18. Degallier N, Sa GC, Monteiro HAO, Castro FC, Da Silva OV, Brandao RCF, et al. Release-recapture experiments with canopy mosquitoes in the Genera *Haemagogus* and *Sabethes* (Diptera : Culicidae) in Brazilian Amazonia. Journal of Medical Entomology. 1998 Nov;35(6):931–6.

19. Day J, Edman J, Scott, T. Reproductive Fitness and Survivorship of *Aedes aegypti* (Diptera: Culicidae) Maintained on Blood, with Field Observations from Thailand. J Med Entomol. 1994;31(4):611–7.

20. Davidson, J, Sudirman R, Wahid I, et al. Mark-release-recapture studies reveal preferred spatial and temporal behaviors of *Anopheles barbirostris* in West Sulawesi, Indonesia. Parasites & Vectors. 2019;12:385.

21. Constantini C, Song-Gang L, Tourre A, et al. Density, survival and dispersal of *Anopheles gambiae* complex mosquitoes in a West African Sudan savanna village. Medical and Veterinary Entomology. 1996;10:203–19.

22. Cianci D, Van den Broek J, Caputo B, Marini F, Della Torre A, Heesterbeek H, et al. Estimating Mosquito Population Size From Mark-Release-Recapture Data. Journal of Medical Entomology. 2013 50(3):533–42.

23. Charlwood JD, Smith T, Billingsley PF, Takken W, Lyimo EOK, Meuwissen J. Survival and infection probabilities of anthropophagic anophelines from an area of high prevalence of *Plasmodium falciparum* in humans. B Entomol Res. 1997 87(5):445–53.

24. Buonaccorsi J, Harrington L, Edman J. Estimation and comparison of mosquito survival rates with release-recapture-removal data. J Med Entomol. 2003;40(1):6–17.

25. Beier JC, et al. Horizontal distribution of adult *Aedes triseriatus* (Diptera: Culicidae) in relation to habitat structure, oviposition and other mosquito species. J Med Entomol. 1982;19(3):239–47.

26. Macdonald, W. A mark-release-recapture experiment with *Mansonia* mosquitos in Malaysia. Southeast Asian J Trop Med Public Health 1990 21(3):424-9.
